# Everybody's Page

**Published:** 1890-03-08

**Authors:** 


### Everybody's Page.

For chapped hands and roughness of skin glycerine and eau de Cologne mixed is one of the most efficacious remedies.

Some good American, with a love for his fellow-creatures, is said to be about to introduce a Bill which shall compel chemists to print on the labels of bottles containing poison, the proper antidote, in case of accidents.

Socialism has numerous sympathisers among the undergraduates of the fair sex at Oxford, if it is true that at a recent debate amongst them a motion approving of "the Socialistic tendencies of the present age" was carried by a large majority.

From the investigations by Professors Forster and De Freytag, salting or pickling seems to have very little destructive power on many of the commoner forms of bacilli, which may be found in diseased meat. The bacilli of typhoid, erysipilas, tubercle, and infectious porcine diseases were found alive after having been in pickle two months.

A writer in *Household Words* shows up those members of society who care more for outward appearance than reality. To be accused of Bohemianism in its true sense is no stigma. To care infinitely more to ensure good education for our children than to be fashionable; to live simply, dress plainly, and so be able to afford good teaching, good books, concerts, pictures, in short, to economise bodily luxuries in order to obtain mental riches is the truest sort of economy.

With reference to the article on "Apples as a Medicine" in our issue of the 8th inst., "Medicus" writes to the *Standard* to point out, on the authority of Dr. Denis Dumont, Surgeon-in-Chief of the Hospital Hotel Dieu, at Caen, that in countries where cider is the habitual beverage stone or calculus is unknown, and probably gout also. A series of inquiries instituted among the medical practitioners in Normandy—a great apple country—and more particularly with those who practised in localities where cider was the principal, if not the sole, beverage, brought to light the fact that some had had an experience of forty years without ever meeting with a single case.

The Hypnotic Somnal is getting anything but a favourable reception from Liebreich and others, who pronounce it to be neither safe nor efficacious.

A project is on foot in the United States to pass a law compelling the appointment of medical women to attend to the female insane in the State asylums.

Although it is wiser not to keep eatables for any length of time in nickel vessels, it has been proved that utensils made of nickel are practically harmless, for under the most favourable circumstances the quantity of nickel dissolved is too small to constitute a danger.

Those bacilli again! They have a deal to answer for. It's they who do untold harm to our hair-follicles and make us grow bald, and thereby spoil our personal appearance; but there is a cure which is simplicity itself, so we are told—we must apply cod-liver oil and the juice of onions. Surely the hardiest baccillus would turn tail at this!

The lode of uranium at the Union Mines in Cornwall, which was discovered some months since, was indeed a good find. The market price of the metal is about £2,000 per ton, and this shows how rare it is, and the works are capable of turning out half a ton a-week. It is largely used in the manufacture of glass and porcelain, and other applications are said to be in contemplation, such as substituting it for gold in electroplating.

The meeting of the French Cheap Dwellings Society a few weeks ago was a great success. Dr. Rochard, of the Académie de Médecine, impressed upon the audience a fact which many apathetic people in London would do well to think over. He pointed out that the dens in which poor people are obliged to live, huddled together without water and without light, are the very homes of tuberculosis and epidemics, and that the richer classes of society ought to fight to the utmost the question of sanitary dwellings, with a view to reducing the ravages of disease and death, not from a feeling of charity or philanthropy for their poorer brethren alone, but for their own interests as well.

